# The Posterior Dominant Rhythm Remains Within Normal Limits in the Microgravity Environment

**DOI:** 10.3390/brainsci14121194

**Published:** 2024-11-27

**Authors:** Vasileios Kokkinos, Andreas M. Koupparis, Tomer Fekete, Eran Privman, Ofer Avin, Ophir Almagor, Oren Shriki, Amir Hadanny

**Affiliations:** 1 Department of Neurology, Feinberg School of Medicine, Northwestern University, Chicago, IL 60611, USA; 2 Comprehensive Epilepsy Center, Northwestern Memorial Hospital, Chicago, IL 60611, USA; 3 Cyprus Institute of Neurology and Genetics, Nicosia 2371, Cyprus; andreask@cing.ac.cy; 4 Brain.Space, Tel Aviv 58855, Israel; tomer.fekete@brain.space (T.F.); eran.privman@brain.space (E.P.); amir.hadanny@brain.space (A.H.); 5 Department of Cognitive and Brain Sciences, Ben-Gurion University, Beer-Sheva 84105, Israel; oferavin@post.bgu.ac.il (O.A.); ophiralm@post.gbu.ac.il (O.A.); shrikio@bgu.ac.il (O.S.)

**Keywords:** electroencephalography, posterior dominant rhythm, alpha, microgravity

## Abstract

Background: Electroencephalogram (EEG) biomarkers with adequate sensitivity and specificity to reflect the brain’s health status can become indispensable for health monitoring during prolonged missions in space. The objective of our study was to assess whether the basic features of the posterior dominant rhythm (PDR) change under microgravity conditions compared to earth-based scalp EEG recordings. Methods: Three crew members during the 16-day AXIOM-1 mission to the International Space Station (ISS), underwent scalp EEG recordings before, during, and after the mission by means of a dry-electrode self-donning headgear designed to support long-term EEG recordings in space. Resting-state recordings were performed with eyes open and closed during relaxed wakefulness. The electrodes representative of EEG activity in each occipital lobe were used, and consecutive PDR oscillations were identified during periods of eye closure. In turn, cursor-based markers were placed at the negative peak of each sinusoidal wave of the PDR. Waveform averaging and time-frequency analysis were performed for all PDR samples for the respective pre-mission, mission, and post-mission EEGs. Results: No significant differences were found in the mean frequency of the PDR in any of the crew subjects between their EEG on the ISS and their pre- or post-mission EEG on ground level. The PDR oscillations varied over a ±1Hz standard deviation range. Similarly, no significant differences were found in PDR’s power spectral density. Conclusions: Our study shows that the spectral features of the PDR remain within normal limits in a short exposure to the microgravity environment, with its frequency manifesting within an acceptable ±1 Hz variation from the pre-mission mean. Further investigations for EEG features and markers reflecting the human brain neurophysiology during space missions are required.

## 1. Introduction

The success of manned space missions is inherently highly dependent on the crew’s motor and cognitive performance integrity [1]. Studies have shown that cognitive and motor performance can deviate from baseline during space missions, affecting key skills such as task management [2], attention [3], the subjective perception of time [4], somatic proprioception [5,6], motor speed and accuracy [7,8,9,10]. Such deviations have been responsible for accidents during missions [11] and introduce significant risks for long-term space mission perspectives [12]. Therefore, the need for reliable biomarkers of motor and cognitive performance deviations arises, which can be used in the context of preventive and countermeasure strategies during the mission.

The electroencephalogram (EEG) is one the most common non-invasive clinical means to monitor and record the state of the human brain [13]. The electrical signals of the brain over time represent the post-synaptic activity of populations of neurons residing in the cortex of the hemispheres [14]. Early enough, the EEG demonstrated the potential to discriminate between the two main states of the brain: wakefulness and sleep [15,16]. More specifically, the EEG provided the main criteria for identifying the stages of sleep in both adult and pediatric populations [17,18]. In addition, long before the development of imaging modalities [19], the EEG established clinically validated biomarkers for the diagnosis of serious neurological disorders, such as epilepsy [20] and focal brain abnormalities e.g., tumors [21]. Today, the EEG is central in the neurological clinical setting and used to understand complex neurological conditions with cognitive impairment, such as dementias [22,23], as well as to monitor patients in critical care over long periods of time [24,25].

The interpretation of the EEG that supports the discrimination between physiologic and pathophysiologic conditions of the brain is performed by evaluating 1. background features, that is, the appearance of the EEG baseline [15,26], and 2. foreground features, that is, distinctive waveforms that stand out from the EEG background [16,27]; the latter appearing in two basic forms: waves and oscillations (rhythms). For example, the appearance of spike-wave discharges in the EEG foreground is recognized as the hallmark of an increased risk for epileptic seizures [28,29], while focal slow waves in the EEG background denote underlying focal brain lesions [30]. Typically, deviations from the baseline, such as amplitude asymmetries or significant phase lag when comparing foreground EEG elements between homologous regions of the two hemispheres signify an underlying pathophysiologic condition [31,32]. As each foreground EEG element is associated with specific neuronal networks in the brain [33], such deviations can be associated with impaired integrity of these specific networks and thereby with a higher risk of impairment in the functions they subserve [34]. However, deviations in certain EEG features have also been associated with improved cognitive performance [35], suggesting that clinical correlations are imperative for the evaluation of EEG biomarkers.

The human EEG has been recorded sparsely in several space missions, beginning from as early as the 60 s [36] to assess the risk of alterations of mental and cognitive function during the mission and identify confounding factors that affect cognitive performance [37,38]. Unfortunately, although several studies have already utilized EEG during space missions and parabolic flights in humans [39,40], as well as in primates in orbit [41], including investigations of the sleep state [42,43] and simulated conditions [44,45,46,47], only a handful of them have attempted to establish EEG biomarkers in a quantitative manner [48,49,50,51], with the majority focusing on event-related potentials (ERPs) rather than foreground EEG elements.

Literature reviews have acknowledged that EEG studies in parabolic flights and spaceflights have often been conflicting [52,53,54,55,56,57], without a clear consensus on what EEG element or set of elements can constitute biomarkers of brain function during prolonged spaceflight. For example, a number of studies during spaceflight have reported significant changes in EEG features when astronauts performing visual stimulation tasks, such as connectivity [48] and ERPs [51], associated with decreased performance in visuospatial processing. In contrast, parabolic flight studies reported either no significant EEG changes [2] or EEG changes associated with improvement of cognitive performance [58,59]. Another EEG study performed during parabolic flight reported increased beta EEG power without quantitatively noticeable effects on the motor performance of the crew while performing a motor tracking task [60]. However, a significant reduction in beta band power has been reported in other parabolic flight studies, interpreted to be associated with lower levels of alertness [61].

The single prior work processing EEG at the spectral level used data from the ODISSEA and CERVANTES missions of the European Space Agency to investigate the effects of gravity on the alpha band (~10 Hz) [62]. The authors reported slight changes in the peak frequency and a significant increase in the whole-brain alpha band power during the mission. However, the authors did not distinguish between the finer foreground EEG elements, namely the posterior dominant rhythm (PDR) and the Mu rhythm [63]—oscillations that share the same frequency band but are generated by separate networks under discrete conditions [64,65,66,67,68,69]—and did not account for the normal inter-subject variability of the PDR [70,71], thereby rendering their results difficult to interpret. Foreground elements of the EEG may share the same morphological features and/or the same frequency band but may appear in either different brain states or in distinct locations on the scalp, which suggests that they represent different physiological states and separate functional brain networks. Such an example is the PDR and the Mu rhythm, which share the same alpha band (8–11 Hz). However, 1. The PDR appears exclusively during eye closure [69] while the Mu rhythm appears during voluntary relaxation of the upper and/or lower extremities [64,65]; 2. The PDR is distributed over the posterior quadrant bilaterally [64,67], while the Mu rhythm is often unilaterally localized over the central region, where the primary sensorimotor cortices reside [64,65,66,72]; and 3. The PDR is generated by thalamo–occipital network interactions associated with elementary and complex vision processing (visuomotor control and object/face recognition) [73,74,75,76,77], while the Mu rhythm is generated by the separate thalamo–somatosensory and sensorimotor connections [78,79] that integrate elementary motor commands as part of the motor control system of the brain [80].

Motivated to provide a clearer account of PDR-only features during spaceflight and further investigate the potential validity of foreground EEG biomarkers for prolonged space missions, we chose to begin by analyzing potential changes and establish variations in key neurophysiological features of the PDR: the first acknowledged EEG rhythm [13]. We selected to quantify and analyze EEG features that are also used for PDR evaluation in clinical settings worldwide, in order to link our findings to the relevant clinical literature. To perform this study, we used an EEG headset specifically designed for microgravity conditions and recorded EEG from crew members of the AXIOM-1 mission to the International Space Station (ISS) in three phases: before, during, and after the mission.

## 2. Materials and Methods

### 2.1. Subjects

The EEG recordings analyzed in this study were performed during the AXIOM-1 mission to the ISS, which took place between 8 and 25 April 2022. Three (3) of the crew members underwent scalp EEG recordings before (18, 17, and 9 days before launch), during (mission days 5, 7, and 10), and after the mission (0, 1, and 2 days after landing). Pre- and post-mission recordings were performed in an office setting at room temperature at variable times during the daytime. None of the crew members experienced significant health impacts and no vision-related issues were reported. All crew members who participated in this study signed an informed consent, and the study was approved by the National Aeronautics and Space Administration (NASA) Institutional Review Board on 13 October 2021 (Study #00000405) and the Northwestern University Institutional Review Board (STU00219484).

### 2.2. Electroencephalography

**Figure 1:**
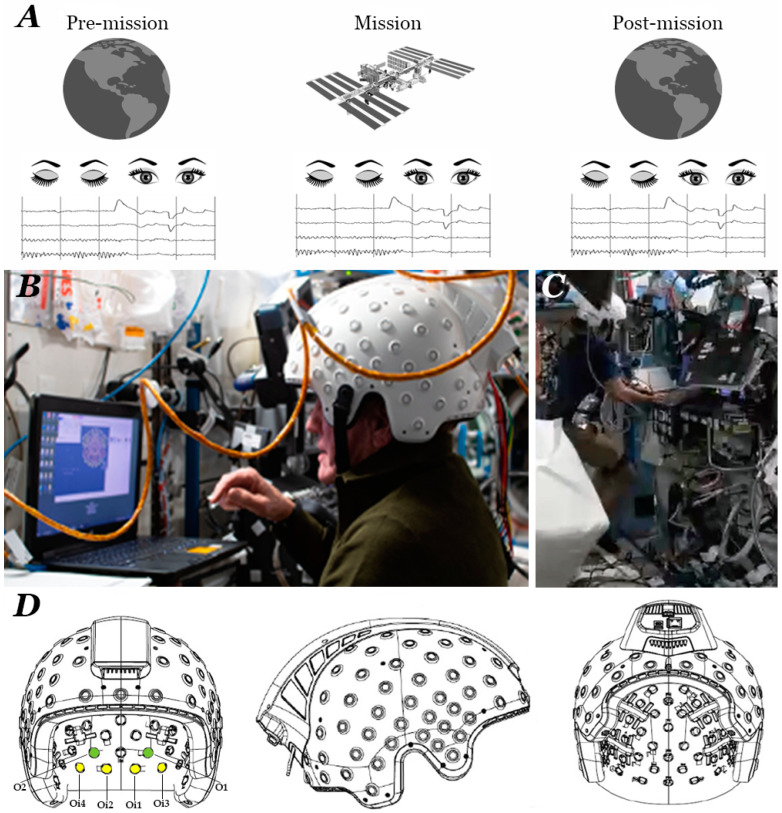
EEG recording protocol, setup, and gear at the International Space Station. (**A**) Schematic of the implemented task to generate reactive PDR before, during, and after the mission to the ISS. The crew member participants performed an eyes-open/eyes-closed task that brought up the PDR during eye closure. (**B**) AXIOM-1 crew member wearing the Brain Sensei™ EEG headgear (brain.space, Tel Aviv, Israel) in front of a laptop computer running brain.space proprietary software that provides step-by-step instructions for efficient donning of the EEG headset in the ISS microgravity environment. (**C**) Body posture of an AXIOM-1 crew member participant while performing research-related tasks with concurrent EEG recording. (**D**) Front, side, and back views of Brain Sensei™ technical drawings. On the left, the position of the occipital electrodes used in this study are shown: O1 and O2 in green, auxiliary Oi1, Oi2, Oi3, and Oi4 in yellow.

All EEG recordings, including the ones at the ISS, were performed by means of the Brain Sensei™ headgear (brain.space, Tel Aviv, Israel) (Figure 1A). The Brain Sensei™ is a battery-powered dry-electrode headset with a pneumatic (pressurized air) sensor positioning system (not shown) that firmly attaches the dry electrodes to the subject’s scalp and makes EEG in reduced gravity conditions possible. It is wired and lightweight (1.65 kgr). It comprises 115 dry EEG brush sensor electrodes placed in positions according to the International 10-5 System, six ground electrodes placed on the forehead and mastoids, and one reference electrode attached to the right earlobe. The system samples the EEG at 500 Hz with analog to digital conversion at 24 bits. Each electrode comprises four (4) brush bundles, and the resulting EEG signal is the averaged signal of these four dry probes. Donning was performed manually by the astronaut in front of the laptop screen running the proprietary software, Sensei™ (version 1.3). The astronaut added pressure until high signal quality markers were shown on the screen. Once donning was completed, the task was initiated on the screen and the astronaut was requested to relax with eyes open for 3 min followed by eyes closed for 3 min. This elementary task typically elicits reactive PDR on EEG whilst the eyes are shut. Following completion of the task, pneumatic pressure was released manually by the astronaut followed by removal of the headgear.

### 2.3. Analysis and Statistics

For each astronaut, EEG recordings were separated into three distinct categories: pre-mission, mission, and post-mission. Qualitative evaluation and quantitative analyses were performed per EEG recording phase, thereby representing the PDR features before, during, and after the mission for each subject. All EEG data were processed in MATLAB (The Mathworks, Natick, MA, USA).

The PDR was identified as an EEG oscillation of waxing and waning profile, bilaterally distributed over the posterior quadrant electrodes, manifesting predominantly during intervals of relaxed wakefulness when the subjects kept their eyes closed [67,69]. To facilitate accurate PDR marking, the EEG signal was band-pass filtered at 5–15 Hz, using a 300-order FIR filter. The FIR filter was designed with a least squares algorithm and applied using zero-phase digital filtering in the MATLAB (R2022a) environment. The order of the filter was calculated at 3 times the 500 Hz sampling rate, divided by the low-frequency cut-off of the filter. Furthermore, the MATLAB implementation of zero-phase filtering doubled the filter order, as the filter was applied twice over the EEG signal. We selected the most representative EEG channels used in clinical practice for PDR identification: O1 for left hemispheric and O2 for right hemispheric PDR, where the PDR typically manifests maximum amplitude. No specific artifact removal algorithm was applied to the EEG data. Muscle and movement artifacts affecting the O1 and/or O2 channels were visually appreciated, and the specific intervals were excluded from further analysis. In case either of these channels contained too much artifact infiltration for analysis to be performed reliably (more than 60% of the total recording), we used the immediately adjacent electrodes if presenting with considerably less artifact (60% or less); that is, electrodes Oi1 or Oi3 and Oi2 or Oi4 to represent, respectively, the left and right occipital regions (Figure 1B). The generic designation O_L_ and O_R_ will be used in the context of this study to represent the left and right hemispheric occipital channels, respectively. The band-passed EEG was in turn reviewed by a board-certified neurophysiologist (V.K.) and a board-certified neurologist (A.M.K.) for PDR elements over the artifact-free occipital channels, and cursor markers were manually placed over each negative peak of the acknowledged PDR oscillations.

**Figure 2:**
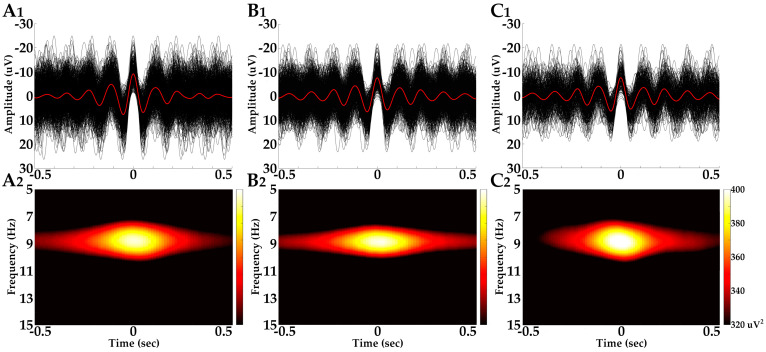
Posterior dominant rhythm (PDR) waveform averages and time-frequency plots from subject 3 recorded from O_L_ territory. (**A_1_**,**A_2_**): Waveform average (red line), overlaid on raw single samples (black lines), and time-frequency plot of pre-mission PDR. (**B_1_**,**B_2_**): Waveform average, overlaid on raw single samples, and time-frequency plot of PDR recorded during the mission to the ISS. (**C_1_**,**C_2_**): Waveform average, overlaid on raw single samples, and time-frequency plot of post-mission PDR. The PDR was reactive to eye closure, with symmetric distribution between hemispheres, and appears to retain its smooth sinusoidal morphology in all three recording phases.

For the qualitative evaluation of the PDR in the time domain, we used all PDR markers placed over every single negative peak of the PDR oscillations. Specifically, for astronaut subject 1, we marked 1138 PDR peaks in O_L_ and 885 PDR peaks in O_R_ from the pre-mission recordings, 445 PDR peaks in O_L_ and 317 PDR peaks in O_R_ from the mission recordings, and 692 PDR peaks in O_L_ and 674 PDR peaks in O_R_ from the post-mission recordings. For astronaut subject 2, we marked 761 PDR peaks in O_L_ and 759 PDR peaks in O_R_ from the pre-mission recordings, 429 PDR peaks in O_L_ and 512 PDR peaks in O_R_ from the mission recordings, and 682 PDR peaks in O_L_ and 572 PDR peaks in O_R_ from the post-mission recordings. For astronaut subject 3, we marked 779 PDR peaks in O_L_ and 903 PDR peaks in O_R_ from the pre-mission recordings, 861 PDR peaks in O_L_ and 902 PDR peaks in O_R_ from the mission recordings, and 426 PDR peaks in O_L_ and 421 PDR peaks in O_R_ from the post-mission recordings. In turn, we performed waveform averaging within a ±0.5 s window from the marker over the PDR negative peaks (0 s). Each manually placed marker was computationally moved to the most negative point of the PDR to increase the accuracy of the analysis. In turn, samples of the same EEG recording phase were superimposed in the ±0.5 s time window, time-locked to the marked negative PDR peaks. The averaged oscillatory waveform was calculated for each EEG recording phase and superimposed over the individual samples for visual appreciation purposes (Figure 2A_1_,B_1_,C_1_).

**Figure 3:**
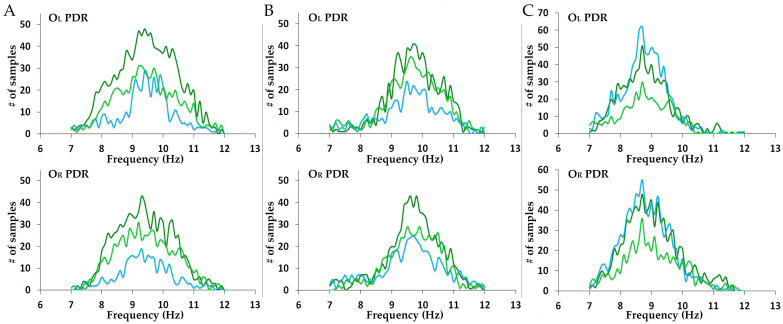
Posterior dominant rhythm (PDR) histograms for all three crew members before, during, and after the mission. (**A**) The amount of individual PDR peaks corresponding to frequencies of oscillation for the left (up) and right (down) occipital region for subject 1 in all three mission phases. (**B**) The amount of individual PDR peaks corresponding to frequencies of oscillation for the left (up) and right (down) occipital region for subject 2 in all three mission phases. (**C**) The amount of individual PDR peaks corresponding to frequencies of oscillation for the left (up) and right (down) occipital region for subject 3 in all three mission phases. Pre-mission values in dark green line, mission values in blue, post-mission values in light green. O_L_ and O_R_ represent left and right hemispheric occipital channels, respectively.

For the quantitative analysis, we used distinct PDR blocks; the latter defined as PDR oscillations of >0.5 s up to 3 s in duration, often presenting with a waxing and waning profile, resulting in PDR peaks maximizing at and around the middle of the block. A single cursor marker was placed over the maximum negative peak for each distinct PDR block. PDR oscillations briefer than 0.5 s and transient PDR waves of the EEG background were not considered for this analysis. For astronaut subject 1, 113 PDR blocks in O_L_ and 88 PDR blocks in O_R_ from the pre-mission recordings, 44 PDR blocks in O_L_ and 31 PDR blocks in O_R_ from the mission recordings, and 69 PDR blocks in O_L_ and 67 PDR blocks in O_R_ from the post-mission recordings were marked. For astronaut subject 2, 76 PDR blocks in O_L_ and 76 PDR blocks in O_R_ from the pre-mission recordings, 42 PDR blocks in O_L_ and 51 PDR blocks in O_R_ from the mission recordings, and 73 PDR blocks in O_L_ and 57 PDR blocks in O_R_ from the post-mission recordings were marked. For astronaut subject 3, 80 PDR blocks in O_L_ and 94 PDR blocks in O_R_ from the pre-mission recordings, 86 PDR blocks in O_L_ and 97 PDR blocks in O_R_ from the mission recordings, and 42 PDR blocks in O_L_ and 42 PDR blocks in O_R_ from the post-mission recordings were marked. The frequency and spectral power analysis of the PDR was performed by means of a short Fast Fourier Transform (FFT). Spectral power was assessed by means of the power spectral density (PSD) metric, which represents the logarithmic (dB) magnitude of the EEG signal energy over the selected interval. We generated 2-D FFT plots (x: frequency; y: PSD) and 3-D time-frequency plots (x: time; y: frequency; z: PSD) from the band-pass filtered and PDR-marked raw data. The FFT settings included a 256-point window with a 250-point sliding overlap, for frequencies from 5 Hz to 15 Hz at a step of 0.05 Hz. 2-D FFT plots were generated for each EEG sample with the marked maximum PDR peak of each discrete oscillation block within a ±0.5 s window from the marker. The frequency and PSD values at the time of the marked maximum negative peak were extracted for each individual PDR block and were used for further statistical analysis. The 3-D time-frequency plots were performed within the same ±0.5 s window and were, in turn, averaged (Figure 2A_2_,B_2_,C_2_). Histograms of the distribution of PDR block maximum negative peak frequencies (*x*-axis in intervals of 0.1 Hz) for both left and right hemispheres during all three EEG recording phases before, during, and after the mission appear in Figure 3.

Statistical analysis was performed to determine whether differences between EEG-derived features (specifically, the PDR blocks-derived maximum peak frequencies and their respective PSD as described in the previous paragraph) among the three phases (per-mission, mission, and post-mission) for each astronaut subject were statistically significant. Statistical significance was assessed by means of two-tailed two-sample *t*-tests at *p* < 0.05 (IBM SPSS Statistics 28.0) in two pairs: pre-mission versus mission (P_PREvsM_) and post-mission versus mission (P_POSTvsM_). For each recording phase, additional statistical analysis was performed between left and right hemisphere frequency and PSD values (P_LvsR_) for control purposes. Due to the significant variability of PDR across subjects reported in prior literature [70,71], combined with the small sample size of three subjects, we did not perform group-level analysis and were instead limited to within-subject analysis.

## 3. Results

### 3.1. PDR Morphology

Qualitative visual appreciation of the averaged PDR waveforms before, during, and after the mission, showed that the PDR retains its smooth sinusoidal morphology (Figure 2A_1_,B_1_,C_1_), reactive to eye closure and symmetric between hemispheres.

### 3.2. PDR Frequency

For both left and right occipital measurements, the mean PDR frequency of oscillation during the mission remained within the pre-mission and post-mission limits without statistically significant changes (Table 1).

More specifically, for subject 1, the mean PDR frequency did not vary more than 0.12 Hz in the left occipital region (pre-mission: 9.44 ± 0.96 Hz; mission 9.31 ± 1.08 Hz; post-mission: 9.29 ± 1.03 Hz) and no more than 0.15 Hz in the right occipital region (pre-mission: 9.34 ± 0.88 Hz; mission: 9.22 ± 1.04 Hz; post-mission: 9.11 ± 0.94 Hz). These variances in PDR mean frequency were not statistically significant (left: pre-mission vs. mission p [P_PREvsM_] = 0.46; post-mission vs. mission p [P_POSTvsM_] = 0.92|right: pre-mission vs. mission p [P_PREvsM_] = 0.53; post-mission vs. mission p [P_POSTvsM_] = 0.60).

For subject 2, the mean PDR frequency did not vary more than 0.32 Hz in the left occipital region (pre-mission: 9.68 ± 1.03 Hz; mission 9.58 ± 1.18 Hz; post-mission: 9.90 ± 1.08 Hz) and no more than 0.30 Hz in the right occipital region (pre-mission: 9.71 ± 1.00 Hz; mission: 9.64 ± 1.30 Hz; post-mission: 9.90 ± 1.01 Hz). These variances in PDR mean frequency were also not statistically significant (left: P_PREvsM_ = 0.63; P_POSTvsM_ = 0.14|right: P_PREvsM_ = 0.73; P_POSTvsM_ = 0.24).

Finally, for subject 3, the mean PDR frequency did not vary more than 0.14 Hz in the left occipital region (pre-mission: 8.74 ± 0.87 Hz; mission 8.80 ± 0.73 Hz; post-mission: 8.95 ± 0.86 Hz) and no more than 0.11 Hz in the right occipital region (pre-mission: 8.93 ± 1.04 Hz; mission: 8.82 ± 0.89 Hz; post-mission: 8.89 ± 1.17 Hz). These variances of PDR mean frequency were also not found to be statistically significant (left: P_PREvsM_ = 0.78; P_POSTvsM_ = 0.30|right: P_PREvsM_ = 0.43; P_POSTvsM_ = 0.70).

No significant differences in PDR mean frequency were found when comparing corresponding left and right occipital values, in all EEG recording phases (Table 1). The standard deviation (SD) of the mean PDR frequency varied from a minimum of 0.73 Hz to a maximum of 1.30 Hz (overall mean SD: 1.00 Hz). Figure 3 demonstrates the overlap of the PDR oscillation range before, during, and after the mission for both right and left occipital measurements.

### 3.3. PDR Power

For both left and right occipital measurements, the mean PSD of the PDR oscillation during the mission remained within the pre-mission and post-mission limits without statistically significant changes (Table 2). More specifically, for subject 1: left: P_PREvsM_ = 0.13, P_POSTvsM_ = 0.18 | right: P_PREvsM_ = 0.63, P_POSTvsM_ = 0.59; for subject 2: left: P_PREvsM_ = 0.49, P_POSTvsM_ = 0.15 | right: P_PREvsM_ = 0.22, P_POSTvsM_ = 0.38; for subject 3: left: P_PREvsM_ = 0.66, P_POSTvsM_ = 0.51 | right: P_PREvsM_ = 0.08, P_POSTvsM_ = 0.22). Also, no significant differences in PDR PSD were found when comparing corresponding left and right occipital values, in all EEG recording phases (Table 2).

## 4. Discussion

We performed a study to assess the morphological, frequency, and power features of the PDR under microgravity conditions, comparing them to the pre- and post-mission EEG recordings. Three members of the AXIOM-1 mission underwent scalp EEG recordings before, during, and after the mission while performing an eyes-open/eyes-closed task during relaxed wakefulness; a process that typically generates PDR oscillations in the posterior quadrant during eyes closure. Qualitative visual inspection showed that the PDR maintained its moth sinusoidal form during the mission. Quantitative analysis showed no significant differences in the mean frequency of the PDR in any of the crew subjects between the EEG recordings on the ISS and earth-level pre- or post-mission EEG. We established that the PDR may normally vary ±1 Hz from the mean frequency of oscillation. We also found no significant differences in the PDR’s power during all phases. Our findings support a within normal limits PDR in the ISS microgravity environment during the short 16-day exposure of the AXIOM-1 mission.

To the best of our knowledge, there haven’t been many previous studies focused on time and frequency features of EEG rhythms during space missions. The single study [62] that addressed EEG oscillations in the posterior quadrant in a comparative manner reported significant changes in the alpha band [63] of crew members during the mission to the ISS. More specifically, the authors reported a change of ~0.5 Hz in the peak frequency of the alpha band, as well as a statistically significant increase in the alpha band power during the mission. Our study showed that variations within ±1 Hz are present both before and after the mission, thereby rendering such deviations within the normal expected limits during the mission. Our study did not confirm the finding of increased alpha power of the PDR during the mission, however, the previous study took into account both the posterior quadrant PDR [67] and the centro-parietal Mu rhythm [64] in their whole-brain alpha dynamics analysis, thereby rendering the comparison to our findings difficult. It is possible though that the previously reported power increase in the alpha band was not attributed to the PDR but to simultaneous variations of the Mu rhythm. In our study, we avoided Mu rhythm infiltration in our PDR analysis by controlling for the task performed and the electrode localization analyzed. More specifically, the Mu rhythm typically appears maximal over the central regions, covered by electrodes C3 and C4, during a simple motor task where the subjects are asked to relax their upper extremities. On the other hand, the PDR appears maximal over the occipital regions, covered by electrodes O1 and O2, during eye closure. Prior literature investigating motor performance during space missions has demonstrated that crew members have to perform extra efforts in order to develop new motor strategies on board [81]. In other words, the demanding conditions at the ISS may require significant adaptation of the sensorimotor networks of the brain, reflected in the features of the Mu rhythm, but no significant changes in the thalamo–occipital circuits that would have been reflected in the PDR. The validity of this explanation demands further focused research, given that the interpretation of deviations in EEG rhythms can be critical for decision-making during the mission. Nevertheless, an increase in Mu power would only reflect the increased effort of the subject to achieve sensorimotor integration during the mission, rather than an abnormal effect induced by microgravity to the brain.

Another potential contributing factor to the discrepancy between our results and past EEG studies in space is the diversity in technical infrastructure used for EEG recording. During missions in the recent past, the standard EEG recording electrodes were attached to an elastic cap—commonly used in research EEG settings worldwide—that has the following acknowledged shortcomings: 1. requires significant preparation time and usually third-party assistance to complete its setup, 2. requires the manual application of conducting gel through the electrode, 3. the instability of the gel over time can result in variable electrode impedance, thereby affecting the EEG signal’s amplitude and 4. the EEG cap’s elasticity can result in notable variation in electrode positions across EEG sessions. These technical shortcomings may render differences between EEG sessions, for example, EEGs collected in different phases of the mission, difficult to interpret. Recent technological advancements in the fields of dry EEG electrodes and electrode positioning have allowed the development of an EEG headset appropriate for zero gravity conditions (Figure 1). This EEG device has several advantages that have rendered it ideal for this study in the space environment. First, its placement can be quickly and easily performed by the individual, without external assistance, thereby eliminating the need for crew members to develop additional EEG technical skills to conduct the recordings. Second, EEG in anti-gravity conditions is made possible by the embedded air pressure-based pneumatic unit that firmly attaches the dry electrodes to the subject’s scalp, thereby allowing for seamless EEG data collection despite the relative orientation of the subject to gravity’s vector. Finally, the lack of need for conducting gel, in combination with the standardized electrode positioning, minimizes the confounding factors and allows for more reliable comparisons between separate EEG sessions.

Although our study was focused on establishing the characteristics of the PDR in microgravity conditions and developing a workflow pipeline for extracting time and frequency features of this rather focal EEG entity, we envision this as the first step towards introducing the EEG as a brain-health monitoring means for long-term missions in space. In that context, determining the normative values of focally manifesting brain oscillations during wakefulness—such as the PDR, the Mu rhythm, the rhythmic mid-temporal theta of drowsiness, and frontal beta—is imperative. Our PDR analysis showed variations of its mean frequency in an approximate ±1 Hz range, which translates into a safety margin of normal PDR variation and into an alarming condition if PDR frequency values are consistently detected outside that range. We chose to focus our investigation of the PDR features over the O1 and O2 electrode channels, as those are the channels of the 10–20 International System of EEG placement where it is optimally evaluated in the clinical setting and thereby we can link our findings to prior clinical literature. Asymmetries in PDR amplitude and frequency have been reported in acute stroke [82,83,84], therefore we can safely assume that regular EEG monitoring during long-term missions will pick up potential deviations in PDR and generate health-related alarms for intervention [85]; also valuable in the post-mission recovery period. In our study, the absence of significant deviations in PDR features is ascribed to the success of the mission in terms of crew member health integrity.

Our study was limited by occasional artifact contamination in the O1 and O2 channels that prevented us from using them throughout the study and we used neighboring channels instead when the artifact levels were obscuring the underlying PDR. However, the proximity of the neighboring electrode channel, positioned on the EEG headgear according to the denser 10-5 International System, allowed for reliable approximation. Another limitation of our study is the small number of subjects analyzed in this study, which made cross-subject analysis difficult. Unfortunately, the number of crew members participating in such missions is currently limited, in addition to this being the first mission of the AXIOM program. We covered for that limitation by performing detailed waveform sampling from each subject, in order to process a statistically acceptable amount of PDR samples for within-subject analysis. Moreover, our findings appear robust regarding the PDR, but given the small sample size from which they were derived, generalization of our results to other subjects and other EEG entities should not be made without caution. We expect our pilot results to validate the need for EEG biomarker development for monitoring brain health and motivate more consistent EEG recordings during missions to the ISS, which would in turn allow an increase in the subject pool and enhance validation. Finally, our results derived from a mission of rather short intervals and are by no means representative of potential changes in brain waves during prolonged missions in space. However, we are confident that, in combination with earth data, such data 1. can prove to be insightful, if changes in other EEG metrics are found (e.g., from other types of analysis, such as connectivity), and 2. can serve as reference for potential future EEG recordings from missions of longer duration and potentially further from ISS, e.g., Lunar or Mars missions.

## 5. Conclusions

This study investigated the PDR as a focal EEG biomarker of posterior thalamo-cortical network integrity under the microgravity conditions of the ISS and found that it remained within normal limits throughout mission phases. EEG biomarkers with adequate sensitivity to identify such deviations could be used in the context of preventive and countermeasure strategies during space missions. More specifically, EEG biomarkers with adequate sensitivity and specificity to reflect the brain’s health status, as well as the integrity of distinct functional networks, can become indispensable for health monitoring during prolonged missions under microgravity [86]. The EEG has the potential to provide a robust set of quantitative biomarkers that can be used during the mission in space to evaluate the overall state of the brain, as well as the status of individual brain networks that underlie cognitive and motor skills [87,88,89]. Complex network analysis of EEG entities would be required to monitor interactions between brain networks responsible for higher-order cognitive and perceptual integration. This approach of developing EEG biomarkers for monitoring brain health can be extended to cover both the crew’s preparatory and training phases, as well as the post-mission recovery period. More importantly, our approach can become valuable for long-term missions, where combined focal and network analysis of a brief EEG recording could provide a quantitative evaluation of the brain’s physiology in an individualized manner for each crew member and on a daily basis. Upon detection of significant deviations from EEG baseline norms, the system would generate alerts addressed to the medically trained crew members and/or the mission control for further evaluation and action. Through this work, we envision the establishment of quantitative EEG analysis among the standard health monitoring procedures during all phases of currently standard and prospectively long-term space missions.

## Figures and Tables

**Table 1:** Mean frequency of the posterior dominant rhythm (PDR) (SD = standard deviation; f = mean frequency; P_PREvsM_ = *p* value between pre-mission versus mission metrics; P_POSTvsM_ = *p* value between post-mission versus mission metrics; P_LvsR_ = *p* value between left and right hemisphere metrics; D_LvsR_ = absolute mean difference between left and right occipital values; D_PREvsM_ = absolute mean difference between pre-mission and mission values; D_POSTvsM_ = absolute mean difference between post-mission and mission values; N = number of distinct PDR blocks; S1–3: Subjects 1–3; O = occipital; L = left; R = right).

	Metric	Pre-Mission Mean ± SD		D_LvsR_ P_LvsR_	Mission Mean ± SD		D_LvsR_ P_LvsR_	Post-Mission Mean ± SD		D_LvsR_ P_LvsR_	D_PREvsM_ P_PREvsM_		D_POSTvsM_ P_POSTvsM_	
		O_L_	O_R_		O_L_	O_R_		O_L_	O_R_		O_L_	O_R_	O_L_	O_R_
S1		(N = 113)	(N = 88)		(N = 44)	(N = 31)		(N = 69)	(N = 67)					
	f (Hz)	9.44 ± 0.96	9.34 ± 0.88	0.10 ± 0.07 0.4483	9.31 ± 1.08	9.22 ± 1.04	0.09 ± 0.03 0.7193	9.29 ± 1.03	9.11 ± 0.94	0.17 ± 0.08 0.2894	0.12 ± 0.12 0.4632	0.12 ± 0.15 0.5351	0.02 ± 0.05 0.9215	0.10 ± 0.09 0.6037
S2		(N = 76)	(N = 76)		(N = 42)	(N = 51)		(N = 73)	(N = 57)					
	f (Hz)	9.68 ± 1.03	9.71 ± 1.00	0.02 ± 0.02 0.8557	9.58 ± 1.18	9.64 ± 1.30	0.05 ± 0.11 0.8180	9.90 ± 1.08	9.90 ± 1.01	0.00 ± 0.06 1	0.10 ± 0.15 0.6327	0.06 ± 0.30 0.7327	0.32 ± 0.10 0.1420	0.26 ± 0.29 0.2458
S3		(N = 80)	(N = 94)		(N = 86)	(N = 97)		(N = 42)	(N = 42)					
	f (Hz)	8.74 ± 0.87	8.93 ± 1.04	0.14 ± 0.19 0.1976	8.80 ± 0.73	8.82 ± 0.89	0.01 ± 0.16 0.8692	8.95 ± 0.86	8.89 ± 1.17	0.06 ± 0.31 0.7895	0.01 ± 0.11 0.6301	0.11 ± 0.14 0.4327	0.14 ± 0.12 0.3057	0.07 ± 0.27 0.7002

**Table 2:** Power spectral density of the posterior dominant rhythm (PDR) (SD = standard deviation; f = mean frequency; P_PREvsM_ = *p* value between pre-mission versus mission metrics; P_POSTvsM_ = *p* value between post-mission versus mission metrics; P_LvsR_ = *p* value between left and right hemisphere metrics; D_LvsR_ = absolute mean difference between left and right occipital values; D_PREvsM_ = absolute mean difference between pre-mission and mission values; D_POSTvsM_ = absolute mean difference between post-mission and mission values; N = number of distinct PDR blocks; S1–3: Subjects 1–3; O = occipital; L = left; R = right).

	Metric	Pre-Mission Mean ± SD		D_LvsR_ P_LvsR_	Mission Mean ± SD		D_LvsR_ P_LvsR_	Post-Mission Mean ± SD		D_LvsR_ P_LvsR_	D_PREvsM_ P_PREvsM_		D_POSTvsM_ P_POSTvsM_	
		O_L_	O_R_		O_L_	O_R_		O_L_	O_R_		O_L_	O_R_	O_L_	O_R_
S1		(N = 113)	(N = 88)		(N = 44)	(N = 31)		(N = 69)	(N = 67)					
	PSD (uV^2^)	8.87 ± 2.66	8.91 ± 2.31	0.04 ± 0.35 0.9110	8.16 ± 2.53	8.68 ± 2.36	0.52 ± 0.17 0.3706	8.78 ± 2.36	8.42 ± 2.22	0.36 ± 0.14 0.3600	0.71 ± 0.13 0.1300	0.23 ± 0.05 0.6363	0.62 ± 0.16 0.1882	0.26 ± 0.14 0.5983
S2		(N = 76)	(N = 76)		(N = 42)	(N = 51)		(N = 73)	(N = 57)					
	PSD (uV^2^)	5.27 ± 2.26	5.41 ± 2.22	0.14 ± 0.04 0.7006	5.57 ± 2.31	4.92 ± 2.20	0.65 ± 0.11 0.1690	4.91 ± 2.38	5.28 ± 2.07	0.37 ± 0.31 0.3411	0.30 ± 0.05 0.4947	0.49 ± 0.02 0.2233	0.66 ± 0.07 0.1506	0.36 ± 0.13 0.3831
S3		(N = 80)	(N = 94)		(N = 86)	(N = 97)		(N = 42)	(N = 42)					
	PSD (uV^2^)	6.56 ± 2.29	6.06 ± 2.24	0.50 ± 0.05 0.1482	6.40 ± 2.38	6.63 ± 2.25	0.23 ± 0.13 0.5026	6.12 ± 1.99	6.15 ± 1.88	0.03 ± 0.11 0.9436	0.16 ± 0.09 0.6600	0.57 ± 0.01 0.0810	0.28 ± 0.39 0.5117	0.48 ± 0.37 0.2280

## Data Availability

All relevant data will be available upon request after approval from the National Aeronautics and Space Administration (NASA), brain.space, and the Northwestern University Institutional Review Board.
